# Supplementary figures and images for: MiR-26a-5p inhibits GSK3β expression and promotes cardiac hypertrophy in vitro
Source: PeerJ. 2020 Nov 17;8:e10371. doi: 10.7717/peerj.10371 (PMC7678492; doi:10.7717/peerj.10371)

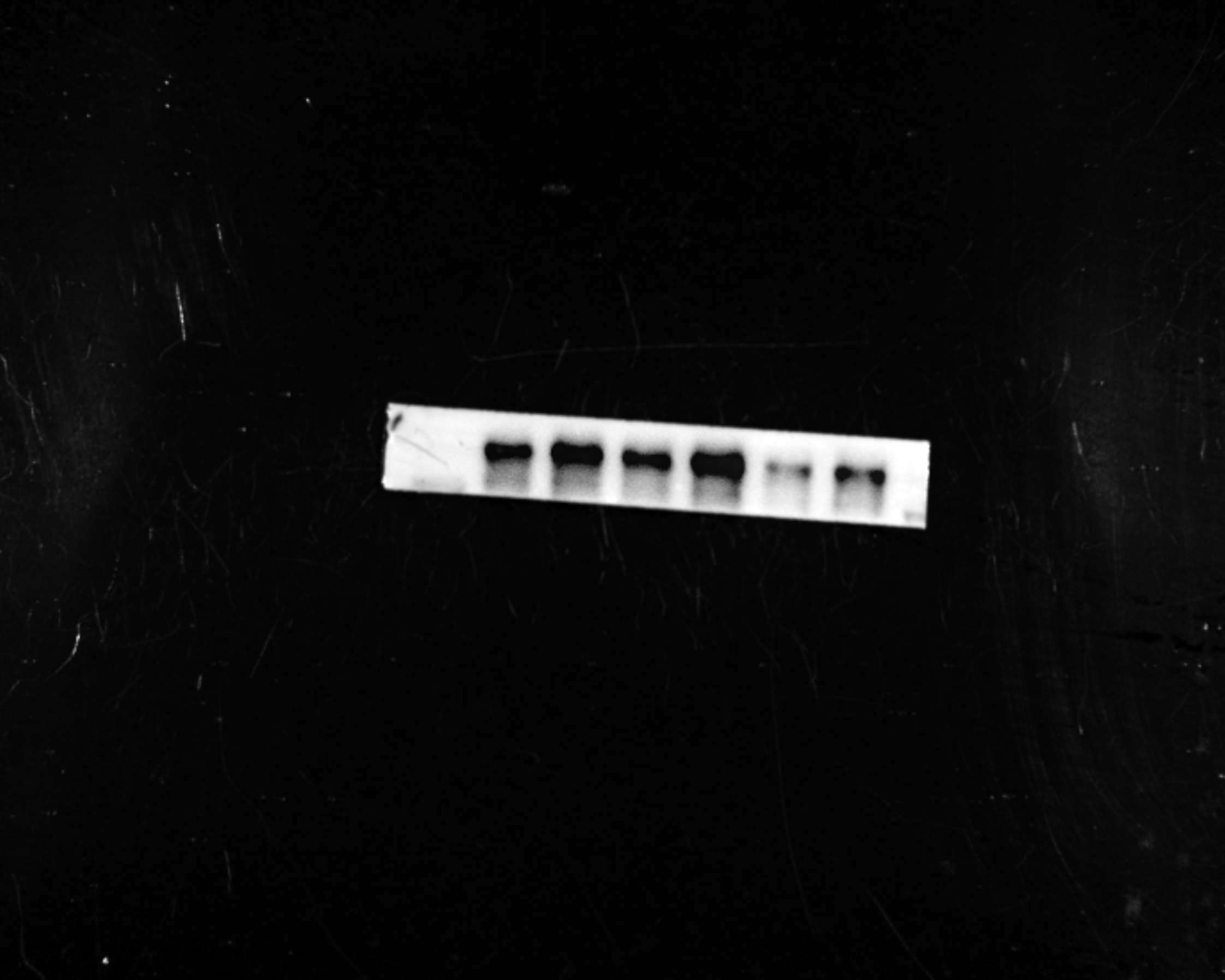

Supplement: Supplemental Information 1 [file peerj-08-10371-s001.zip › Raw data/Figure 1/Figure 1B/Beclin-1.tif]

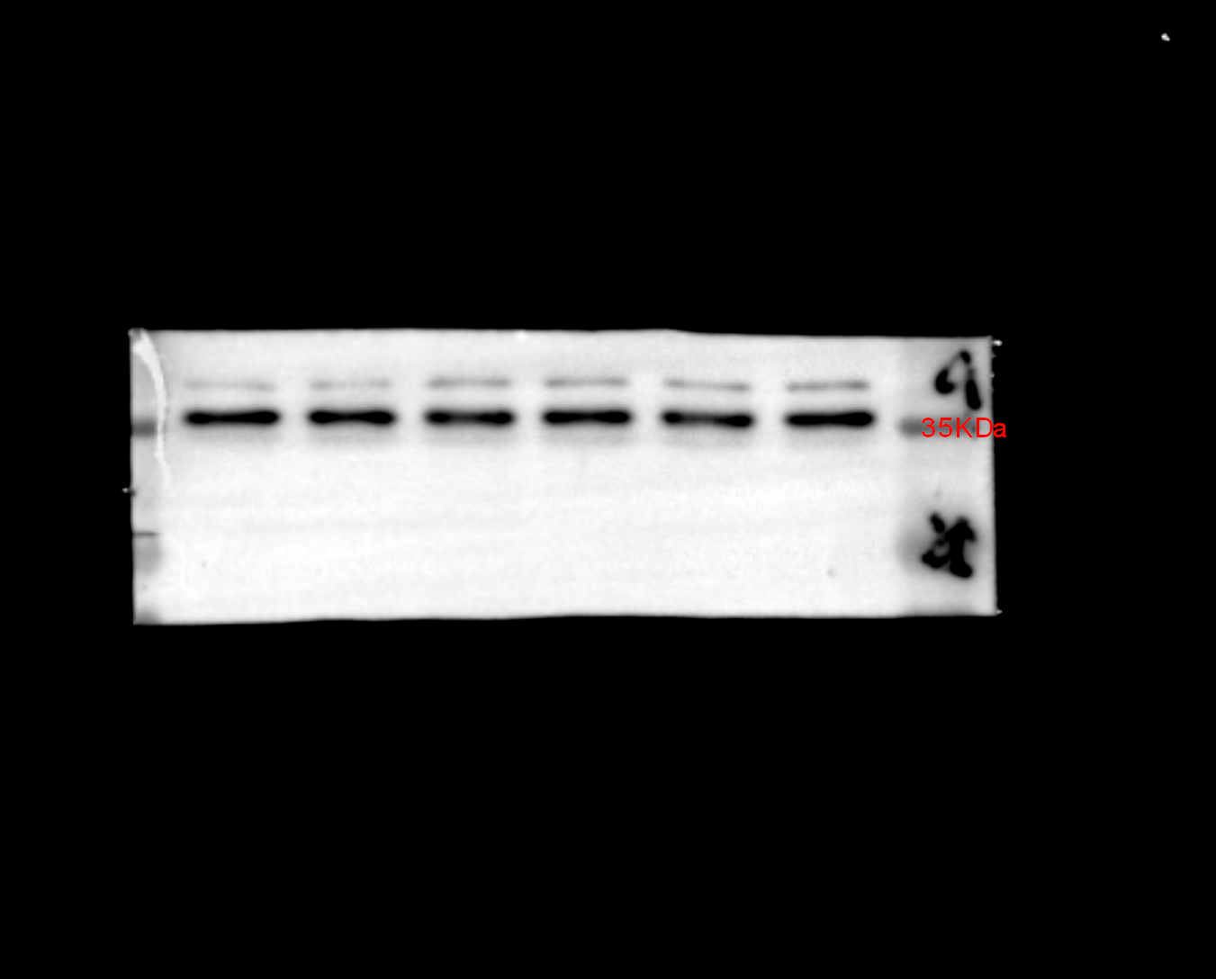

Supplement: Supplemental Information 1 [file peerj-08-10371-s001.zip › Raw data/Figure 1/Figure 1B/GAPDH.tif]

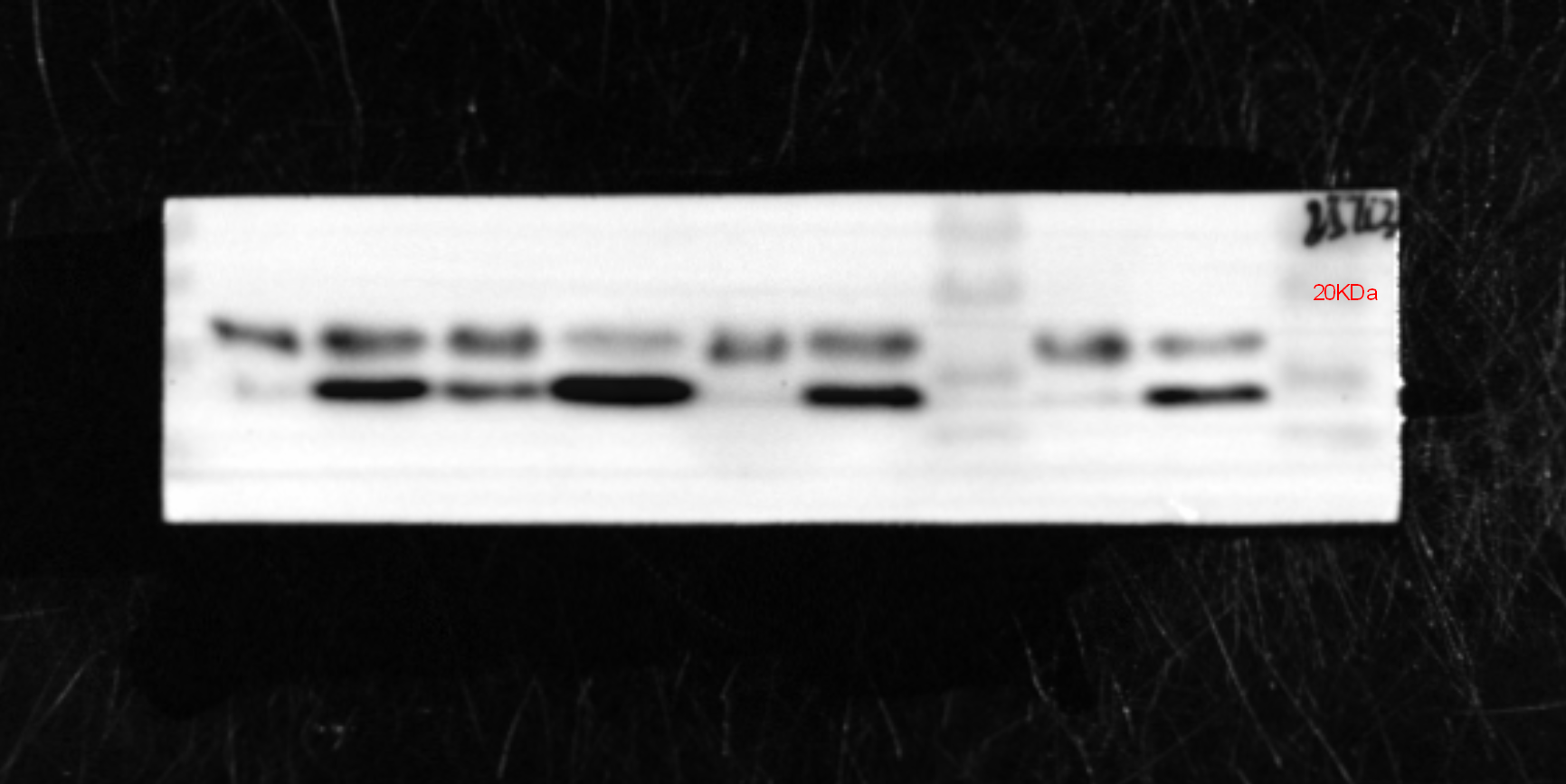

Supplement: Supplemental Information 1 [file peerj-08-10371-s001.zip › Raw data/Figure 1/Figure 1B/LC3.tif]

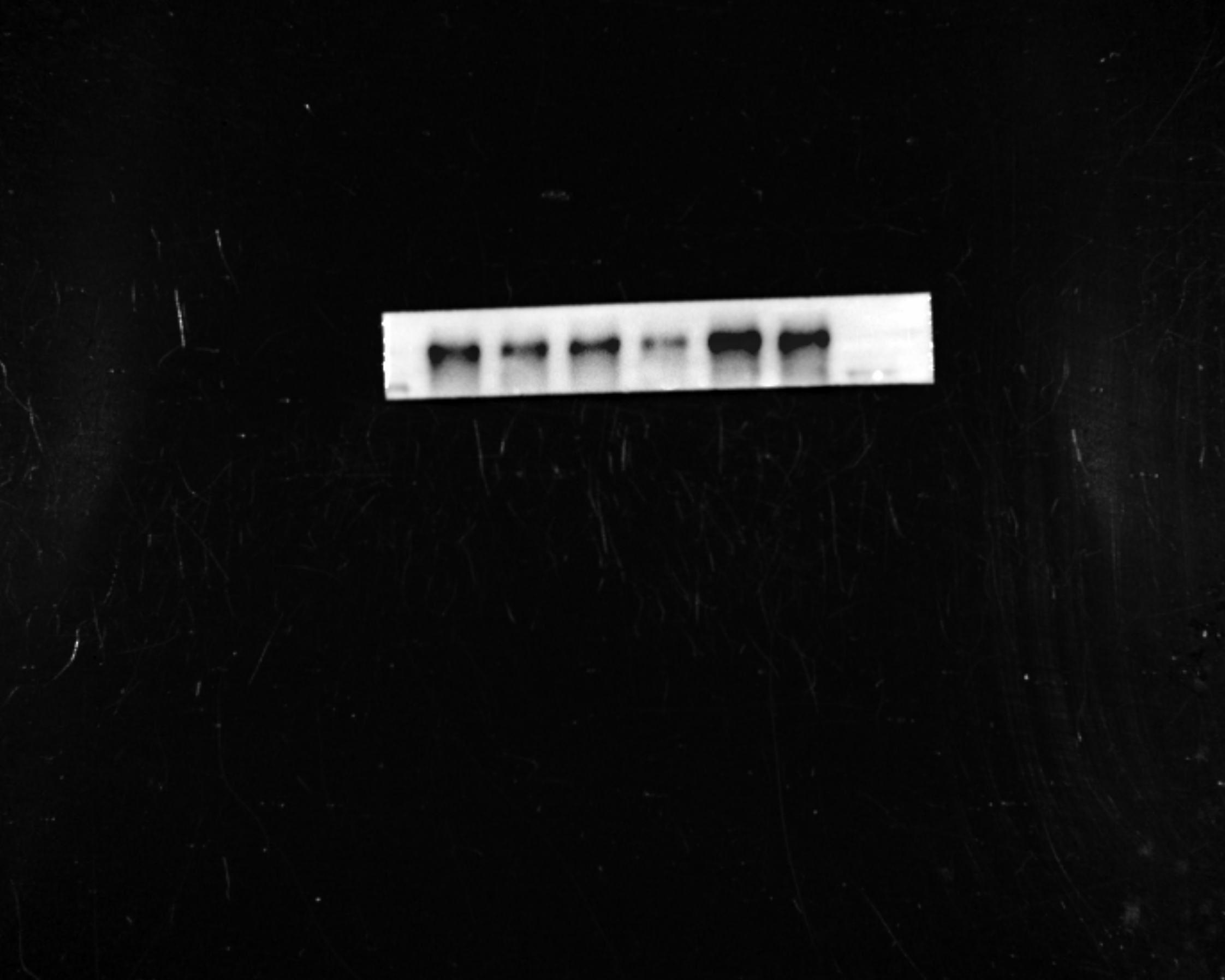

Supplement: Supplemental Information 1 [file peerj-08-10371-s001.zip › Raw data/Figure 1/Figure 1B/p62.tif]

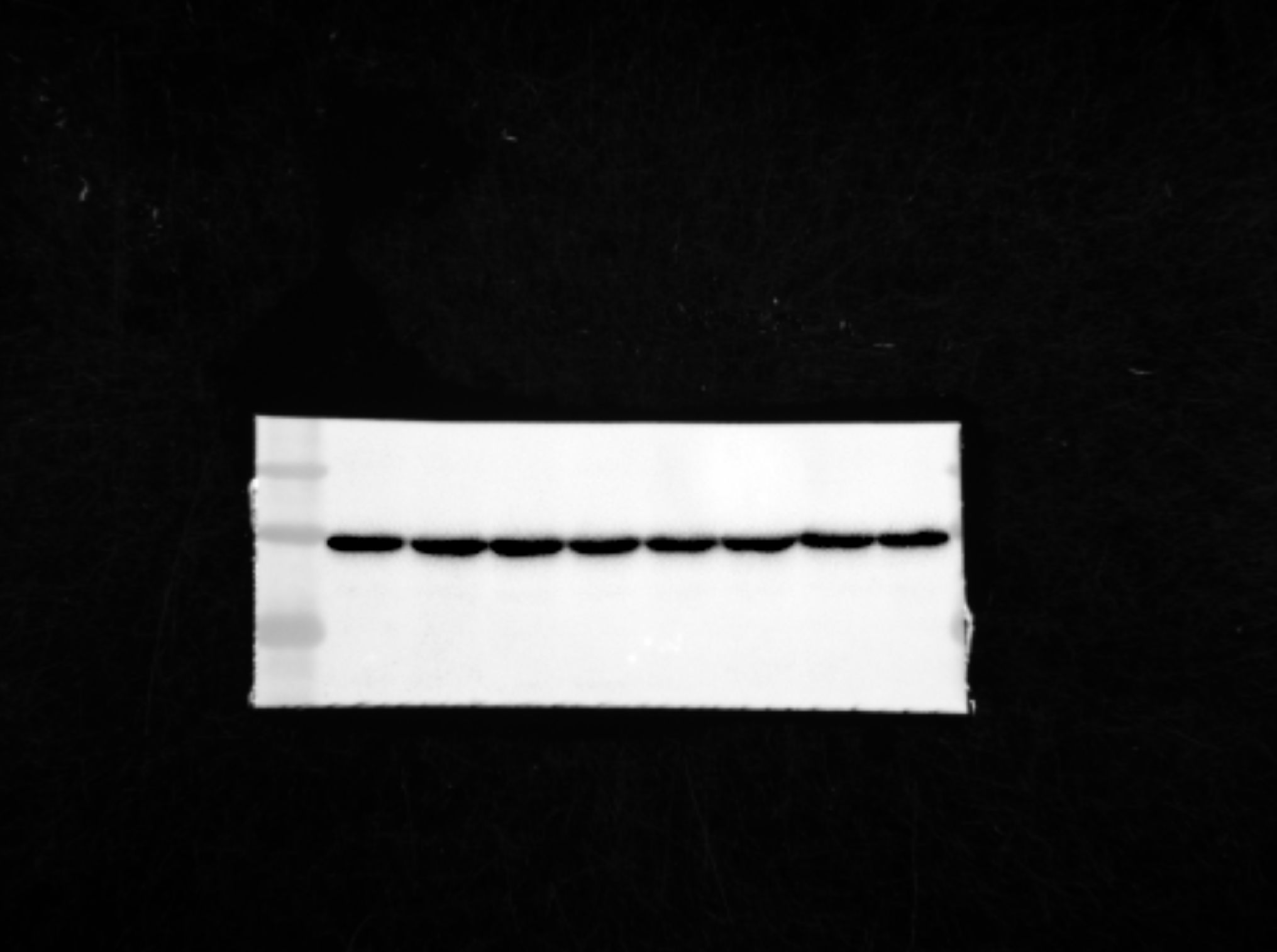

Supplement: Supplemental Information 1 [file peerj-08-10371-s001.zip › Raw data/Figure 1/Figure 1F/GAPDH.tif]

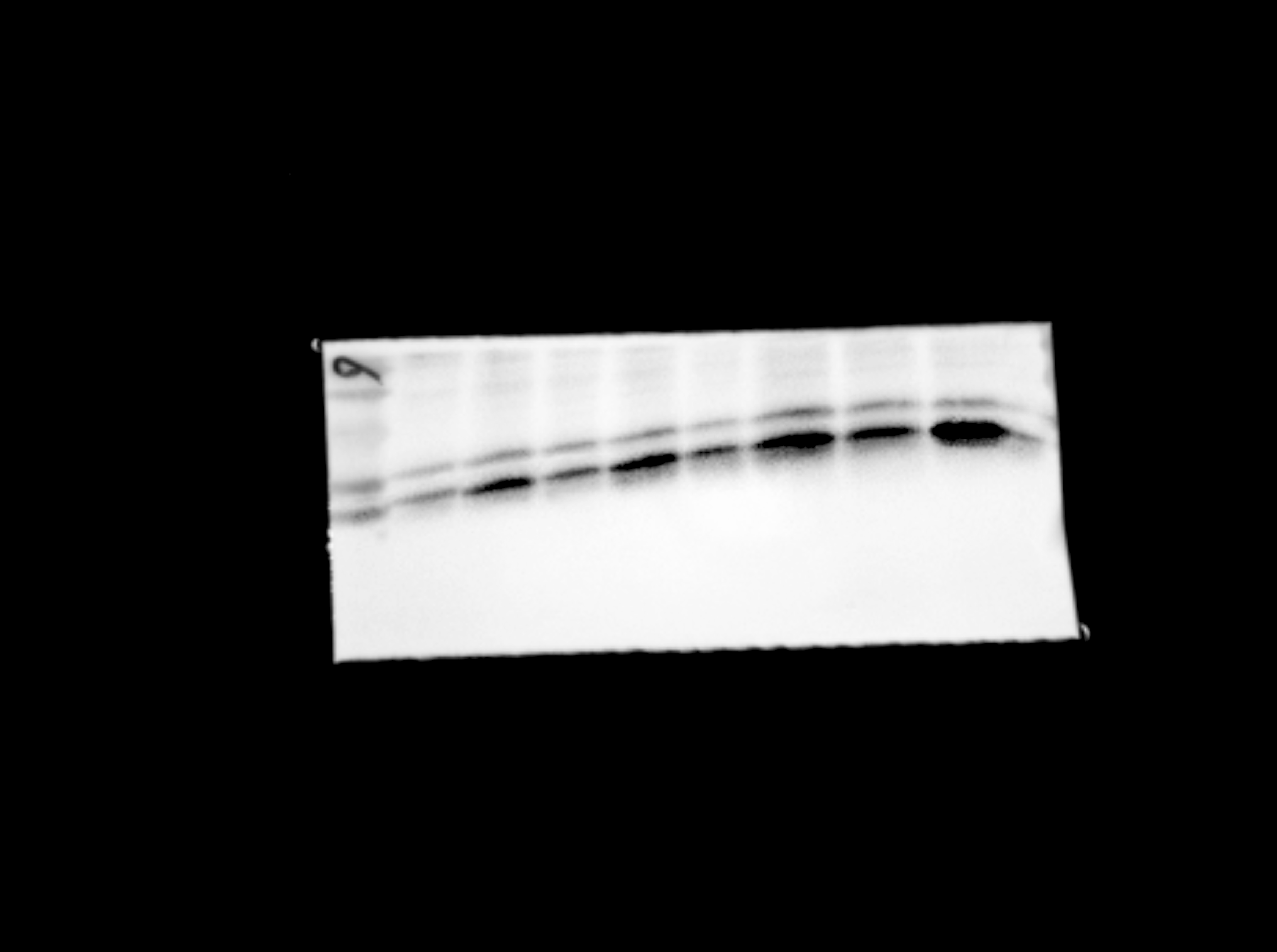

Supplement: Supplemental Information 1 [file peerj-08-10371-s001.zip › Raw data/Figure 1/Figure 1F/LC3.tif]

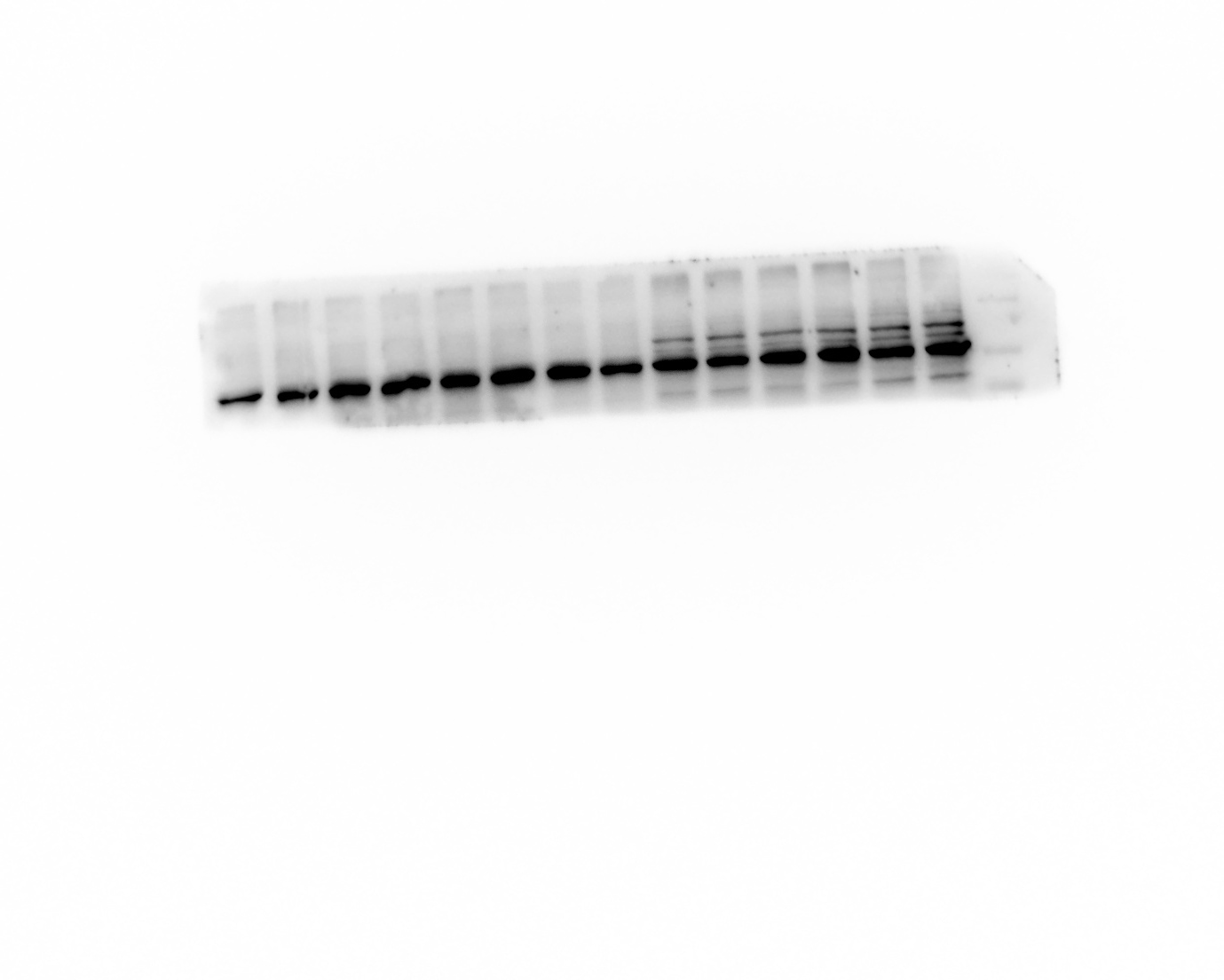

Supplement: Supplemental Information 1 [file peerj-08-10371-s001.zip › Raw data/Figure 1/Figure 1F/p62.jpg]

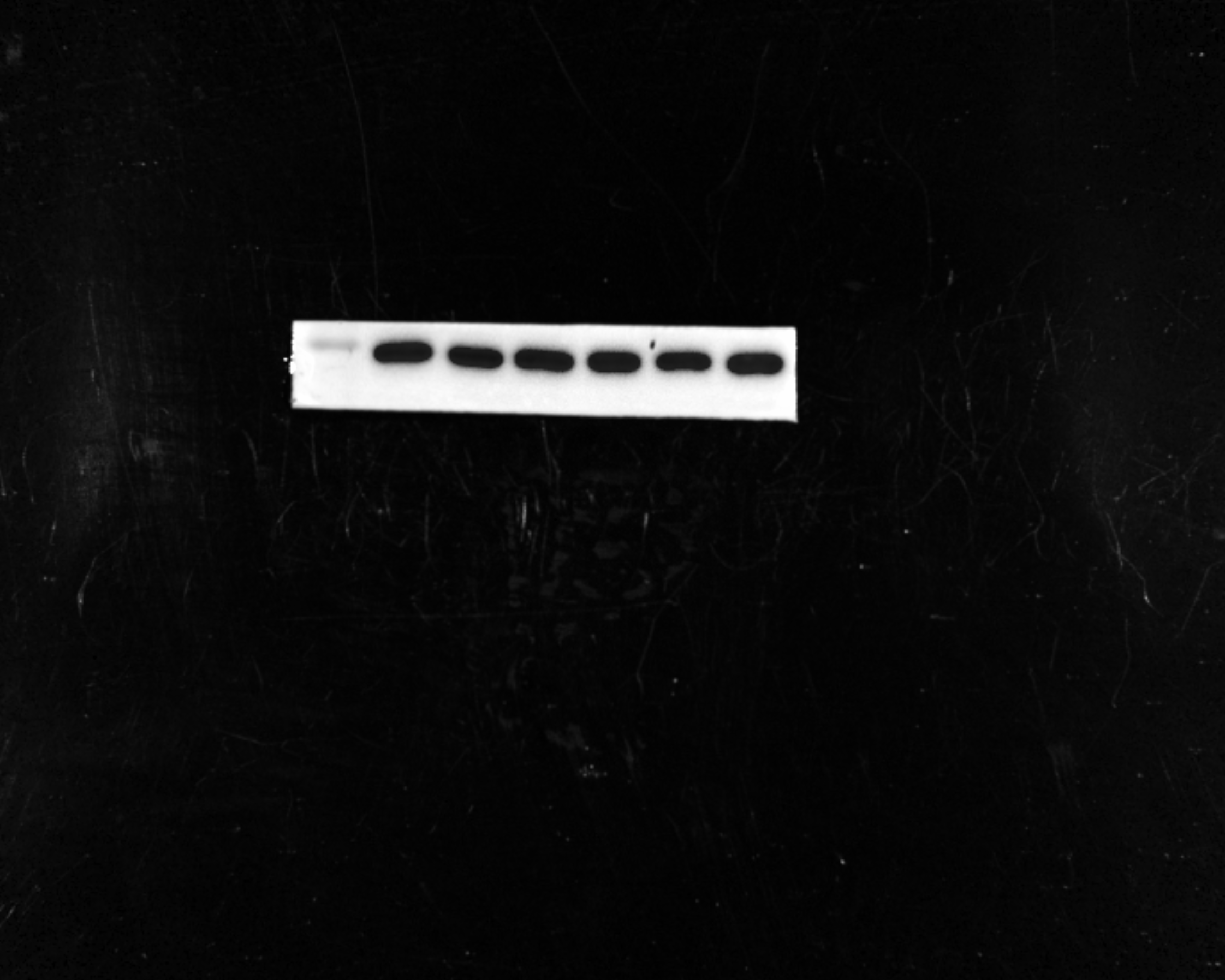

Supplement: Supplemental Information 1 [file peerj-08-10371-s001.zip › Raw data/Figure 3/Figure 3C/GAPDH.tif]

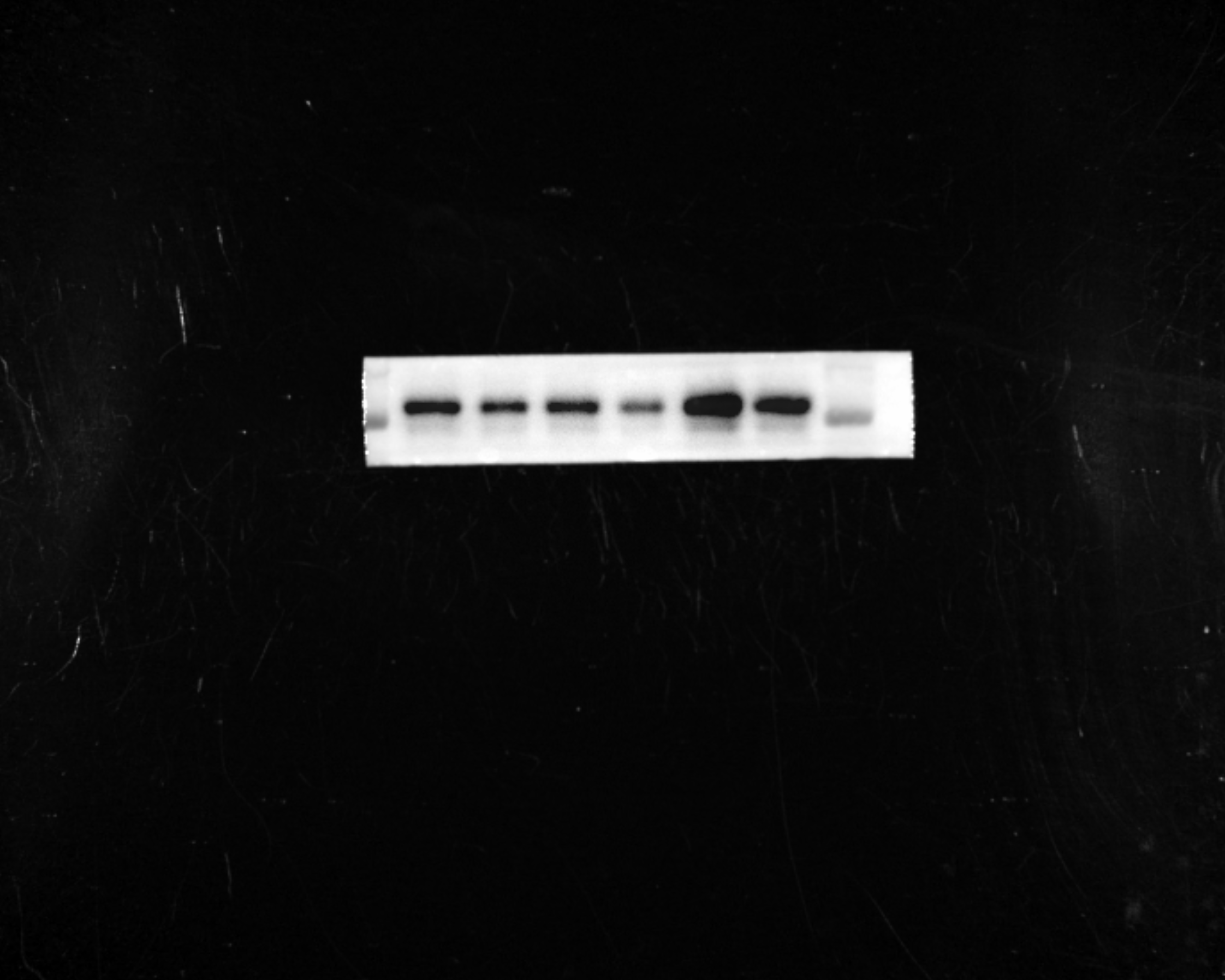

Supplement: Supplemental Information 1 [file peerj-08-10371-s001.zip › Raw data/Figure 3/Figure 3C/GSK3β.tif]

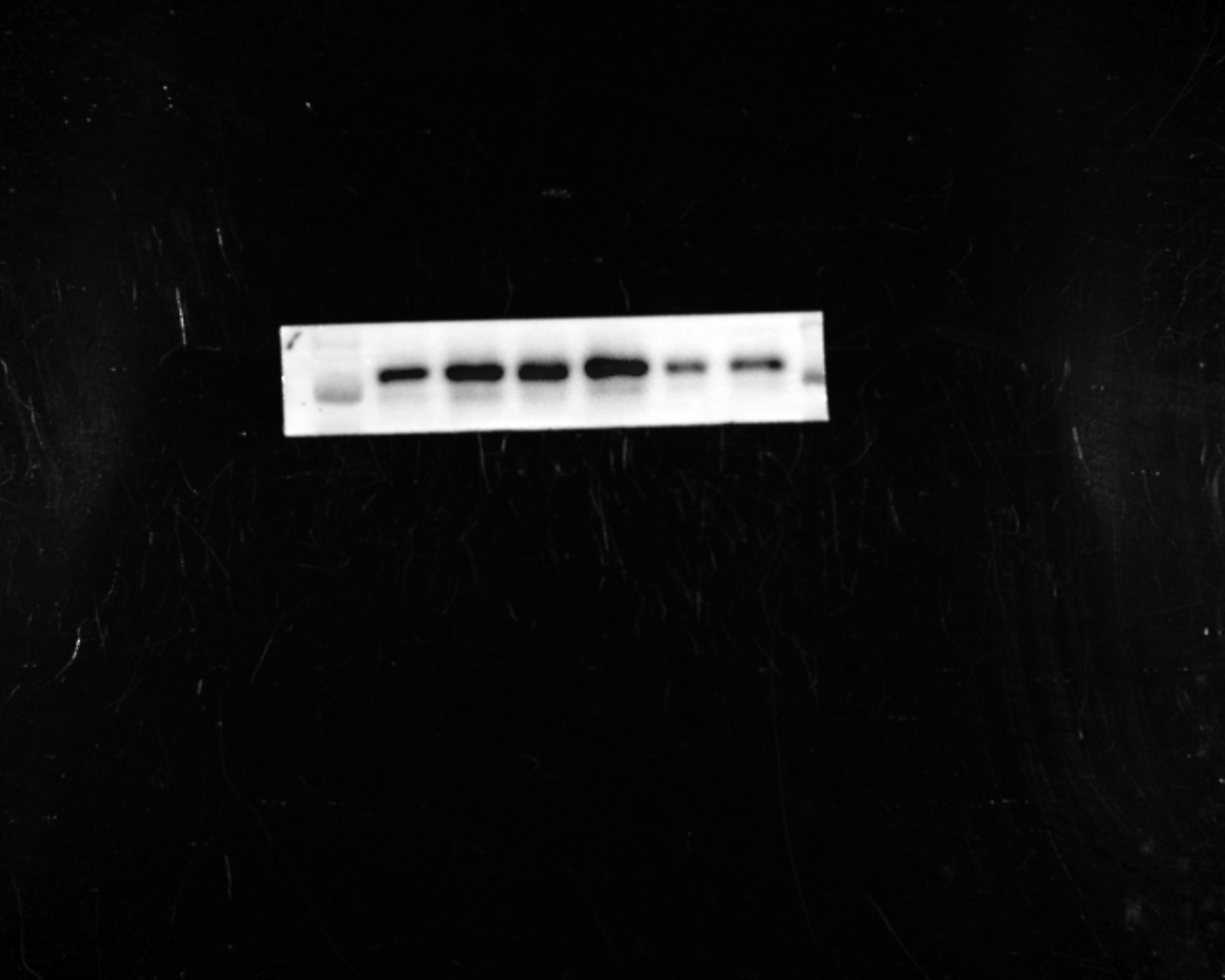

Supplement: Supplemental Information 1 [file peerj-08-10371-s001.zip › Raw data/Figure 3/Figure 3C/α-actinin.tif]

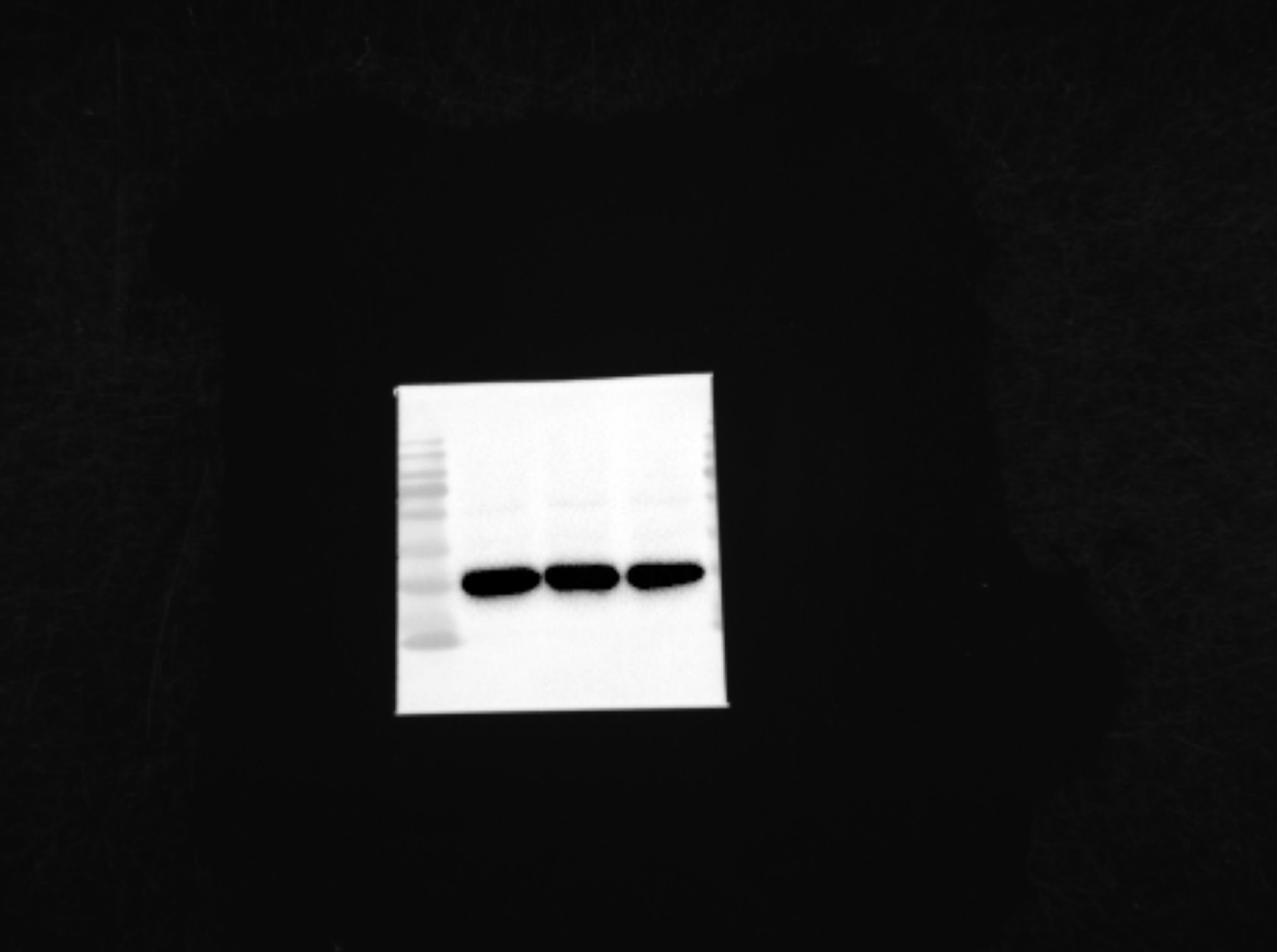

Supplement: Supplemental Information 1 [file peerj-08-10371-s001.zip › Raw data/Figure 6/Figure 6G/GAPDH.tif]

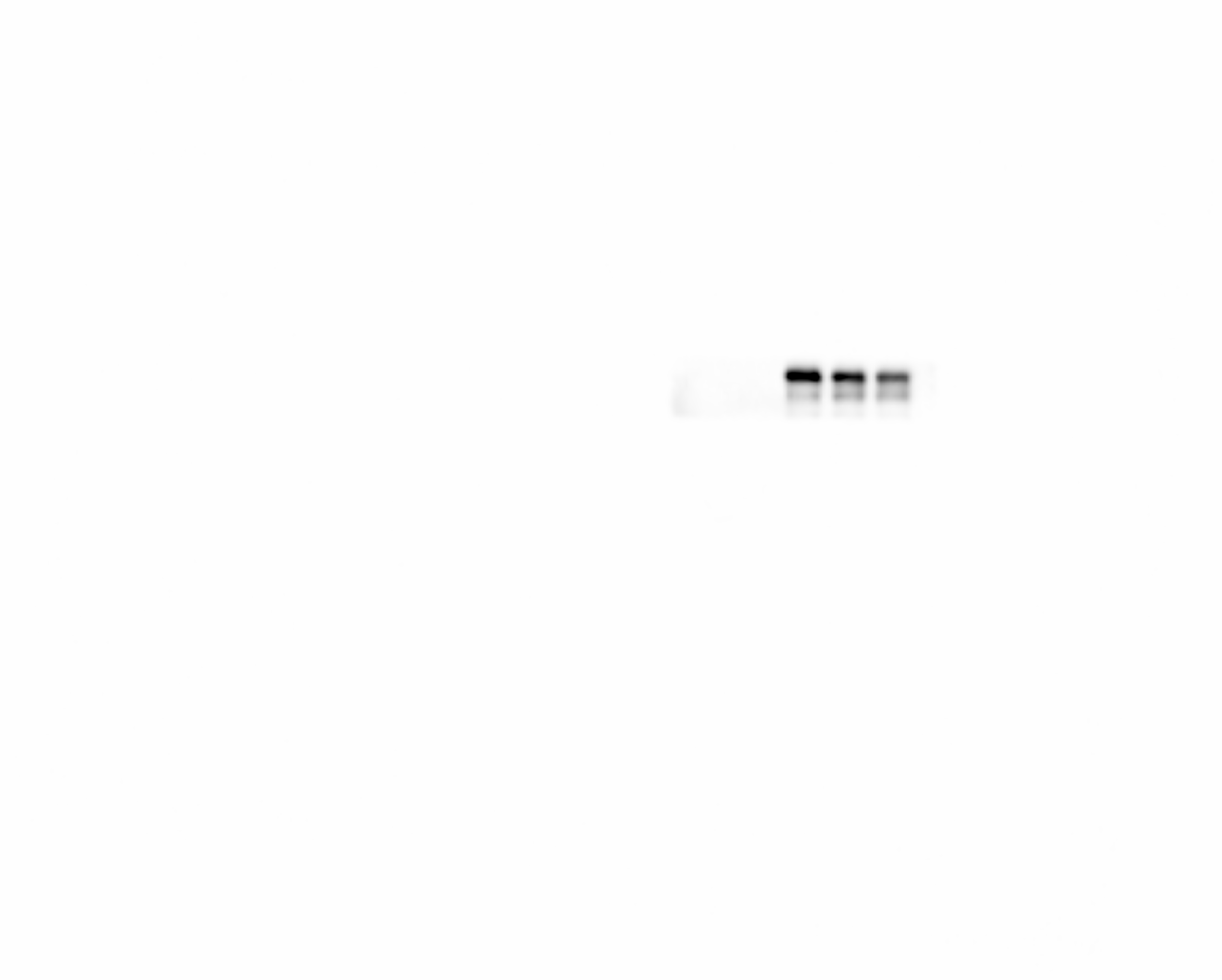

Supplement: Supplemental Information 1 [file peerj-08-10371-s001.zip › Raw data/Figure 6/Figure 6G/GSK3β.tif]

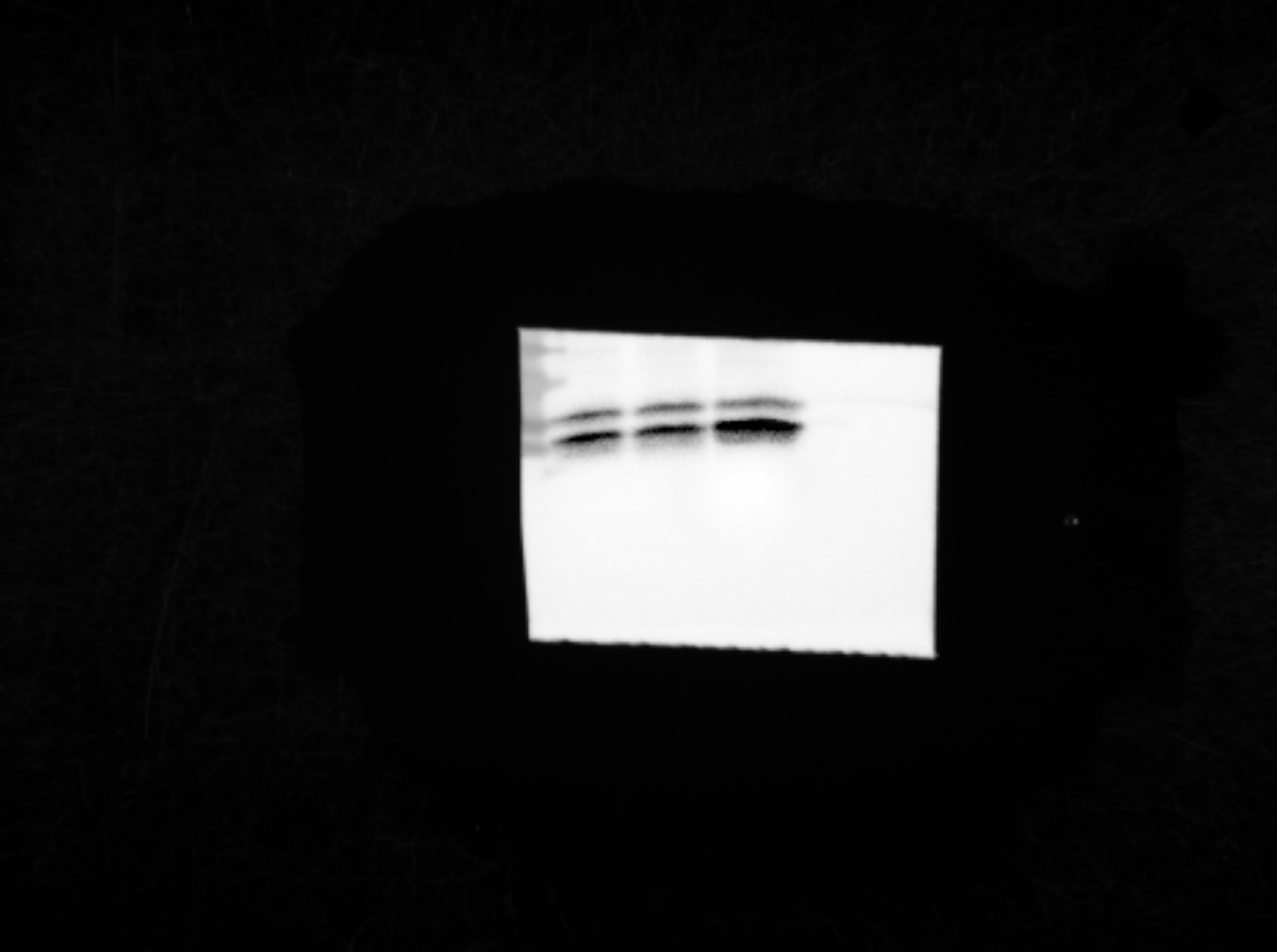

Supplement: Supplemental Information 1 [file peerj-08-10371-s001.zip › Raw data/Figure 6/Figure 6G/LC3.tif]

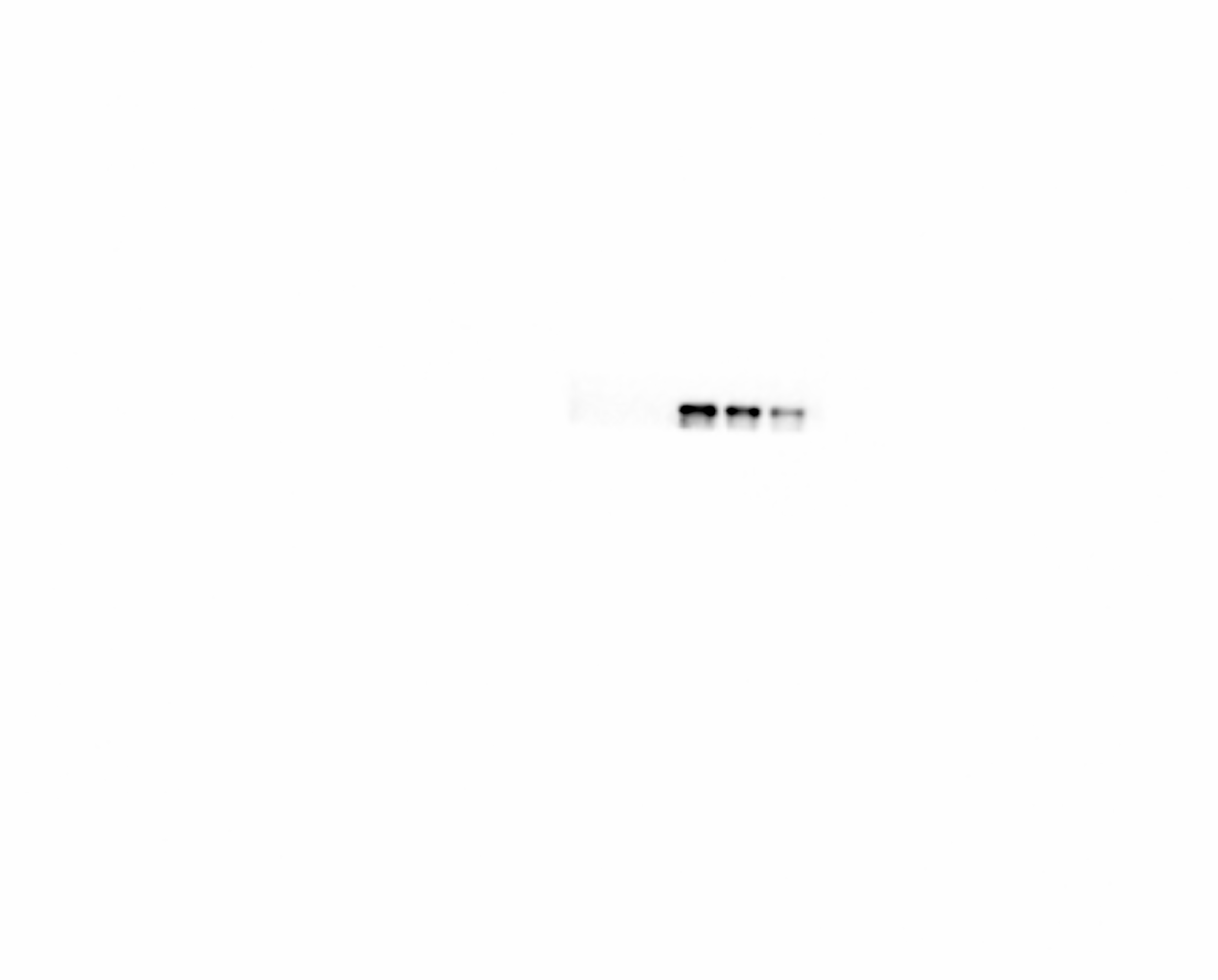

Supplement: Supplemental Information 1 [file peerj-08-10371-s001.zip › Raw data/Figure 6/Figure 6G/p62.tif]
